# Visfatin improves survival and promotes the activation of primordial follicles in cultured sheep ovaries

**DOI:** 10.1590/1984-3143-AR2023-0163

**Published:** 2025-10-17

**Authors:** Joisyleide Gonçalves da Costa Pinto, Ricássio de Sousa Barberino, Valéria da Silva Guimarães, Joãozito Liandro de Oliveira, Alane Pains Oliveira do Monte, Kíscyla Oliveira de Andrade, Maria Helena Tavares de Matos

**Affiliations:** 1 Colegiado de Medicina Veterinária, Universidade Federal do Vale do São Francisco, Petrolina, PE, Brasil

**Keywords:** adipokine, cell proliferation, follicle growth, inflammation, oocyte

## Abstract

Visfatin is an adipokine involved in the regulation of female reproduction. However, there are no studies on the effects of visfatin on the *in vitro* culture of ovarian tissue in any species. Therefore, the aims of this study were to analyze the effects of visfatin on survival, primordial follicle activation, granulosa cell proliferation, and the immunostaining of tumour necrosis factor-α (TNF-α) in preantral follicles after the *in vitro* culture of sheep ovarian tissue. Ovarian fragments were fixed for histological analysis (fresh control) or cultured in α-minimum essential medium alone (α-MEM^+^: control medium) or in α-MEM^+^ supplemented with different concentrations of visfatin (1 or 10 ng/mL) for 7 days. Subsequently, ovarian tissue was destined to histology (morphology, activation and growth) and immunohistochemistry (granulosa cell proliferation and pro-inflammatory cytokine TNF-α immunostaining). The results indicated that treatments with visfatin (1 or 10 ng/mL) maintained the percentage of morphologically normal follicles at a level similar (P>0.05) to the fresh control and significantly higher than of α-MEM^+^. A significant increase in primordial follicle activation was also observed in tissue cultured for 7 days at both visfatin concentrations compared to the fresh control and α-MEM^+^. In addition, only the treatment containing 10 ng/mL of visfatin significantly increased follicular and oocyte diameters, and granulosa cell proliferation compared to α-MEM^+^, and attenuated inflammatory activity by reducing TNF-α immunostaining after *in vitro* culture. In conclusion, 10 ng/mL visfatin maintains survival, reduces immunostainig of TNF-α and promotes the activation of primordial follicles by stimulating granulosa cell proliferation after the *in vitro* culture of sheep ovarian tissue.

## Introduction

*In vitro* culture of ovarian tissue is an important approach to optimize the use of the female germ cell pool by providing a large supply of grown follicles and subsequent mature gametes from primordial follicles, representing a promising method for preserving female fertility (McLaughlin et al., 2018; Costa et al., 2022). Studies have investigated medium supplements, such as antioxidants, growth factors, and hormones, as well as mechanisms that control the *in vitro* activation of quiescent primordial follicles and subsequent follicular growth (bovine: Costa et al., 2022; ovine: Lins et al., 2021; Barberino et al.*,* 2022; Mohammadzadeh et al., 2023).

Visfatin, also known as pre-B cell colony-enhancing factor or nicotinamide phosphoribosyltransferase (Reverchon et al., 2013; Reverchon et al., 2016), is an adipokine produced by oocytes (swine: Mlyczyńska et al.*,* 2023), granulosa (human: Reverchon et al., 2013; bovine: Reverchon et al., 2016; swine: Mlyczyńska et al.*,* 2023) and theca cells (swine: Mlyczyńska et al.*,* 2023) of preantral and/or antral follicles, which demonstrates that visfatin may regulate follicular development. Visfatin is secreted by many other tissues, including adipose, visceral, and subcutaneous tissues (Tanaka et al., 2007; Dahl et al.*,* 2012), uterus (Annie et al., 2019), and placenta (Fasshauer et al.*,* 2007). It is involved in cellular metabolism responses (Rongvaux et al., 2002; Revollo et al. 2007), inflammation, oxidative stress control (Ognjanovic et al., 2005; Friebe et al., 2011), and immune modulation (Moschen et al., 2007).

In an *in vivo* study conducted by Park et al. (2020), visfatin administration in aged mice enhanced preantral follicle development by stimulating the expression of components (4EBP1, S6K1, and RPS6) involved in the phosphatidylinositol 3-kinase/mammalian target of rapamycin (PI3K/mTOR) signaling pathway, which appears to be the primary pathway regulating follicular growth. Furthermore, following superovulation and mating of female mice treated with visfatin, the authors demonstrated that visfatin treatment increased the number of recovered zygotes and the rates of blastocyst formation and embryonic development (Park et al.*,* 2020). Under *in vitro* conditions, gonadotrophins and steroids increased visfatin levels and insulin lowers it, while the action of prostaglandins is dose- and cell type-dependent (swine: Mlyczyńska et al., 2023). Visfatin in association with insulin-like growth factor-1 (IGF-1) increased the secretion of estradiol in isolated granulosa cells (human: Reverchon et al., 2013; bovine: Reverchon et al., 2016) and the release of progesterone in luteal cells (buffalo: Thakre et al.*,* 2021). Nonetheless, there are no studies investigating the effects of visfatin on the *in vitro* activation and development of preantral follicles in any species.

Considering that development of preantral follicles using an *in vitro* culture system would provide a large supply of mature oocytes to be used in assisted reproductive techniques in mammalian species, the aims of this study were to analyze the effects of visfatin on survival, primordial follicle activation, granulosa cell proliferation, and the immunostaining of pro-inflammatory cytokine tumour necrosis factor-α (TNFα) in preantral follicles after *in vitro* culture of sheep ovarian tissue. Sheep are present on all continents and are commercially seen as highly attractive livestock (Gurunathan, 2022). Furthermore, studies with sheep are important to improve our knowledge about the factors that control early folliculogenesis in mammals and to explore possible physiological differences among species (Milenkovic et al., 2011).

## Methods

### Reagents

Alfa-modified minimum essential medium (α-MEM; ref: M8042), visfatin (ref: SRP4908) and other supplements were purchased from Sigma Aldrich Chemical C. (St. Louis, MO, USA). Saline solution, alcohol, formalin, paraffin, and citrate buffer were obtained from Dinâmica (São Paulo, Brazil), and hematoxylin and eosin from QEEL (São Paulo, Brazil). Hydrogen peroxide (H_2_O_2_), 1% normal goat serum, diaminobenzidine (DAB) and EasyLink One Polymer were purchased from EasyPath (São Paulo, Brazil). The antibodies used were anti-proliferating cell nuclear antigen (anti-PCNA; Abcam, Cambridge, MA, USA; ref: ab18197) and anti-tumor necrosis factor-α (anti-TNF-α; Elabscience, Danvers, MA, USA; ref: EAB-33121).

### Source of ovarian tissue

All biological materials used in the study were provided by the local slaughterhouse. The ovaries were collected from already slaughtered animals, therefore ethics committee approval was not required. Ovaries (n=10) from five adult crossbred sheep were collected and washed once in 70% alcohol, and then twice in saline solution supplemented with antibiotics (100 µg/mL streptomycin and 100 µg/mL penicillin). They were then transported to the laboratory at 4ºC (Soares et al., 2018).

### *In vitro* culture of ovarian tissue

*In vitro* culture was conducted following the protocol described by Monte et al. (2021). In the laboratory, following fragmentation of the ovarian cortex using a scalpel under sterile conditions, five slices measuring approximately 3x3x1 mm were obtained. Subsequently, two ovarian fragments from each animal was immediately fixed in 10% buffered formalin for histological analysis and served as fresh control. The remaining three slices of ovarian cortex were randomly distributed into different treatments and individually cultured for 7 days in 1 mL of culture medium in 24-well dishes at 39ºC in a humidified atmosphere of 5% CO_2._ The standard culture medium consisted of α-MEM (pH 7.2 – 7.4) supplemented with 10 ng/mL insulin, 5.5 µg/mL transferrin, 5 ng/mL selenium, 2 mM glutamine, 2 mM hypoxanthine, 1.25 mg/mL bovine serum albumin (BSA), and 50 µg/mL ascorbic acid and then referred as α-MEM^+^ (control medium). To assess the impact of visfatin on the *in vitro* culture of sheep ovarian cortex, the slices were divided into three groups: α‐MEM+ (control medium) or α-MEM^+^ supplemented with 1 or 10 ng/mL visfatin. The media and treatments were replenished every two days. Each treatment was repeated five times (five replicates), thus involving the ovaries of five different animals. Visfatin concentrations were chosen based on a previous study that showed its effects on granulosa cell steroidogenesis and proliferation *in vitro* (bovine: Reverchon et al., 2016).

### Morphological analysis and assessment of *in vitro* follicular activation and growth

The non-cultured fragments (fresh control) and those cultured in control medium or with visfatin were fixed in 10% buffered formalin, cut into 5 µm thick serial sections, mounted on glass slides, and stained with hematoxylin-eosin (HE) for routine histological examination, under a light microscope (Nikon, Tokyo, Japan) at 400x magnification. Preantral follicles were classified as primordial (an oocyte surrounded by one layer of flattened granulosa cells), intermediate (one layer of flattened and cuboidal granulosa cells), primary (one layer of cuboidal granulosa cells), and secondary (two or more layers of cuboidal granulosa cells). They were considered morphologically normal if no overt signs of degeneration were noted, which included a shrunken oocyte, disorganization of the granulosa cell layer, condensed nuclear chromatin, and/or cell swelling (Monte et al.*,* 2021). To avoid double‐counting of the same follicles, each follicle was counted on the first section of the ovarian fragment where the centrally located nucleus of the oocyte appeared. Overall, 150 follicles were evaluated for each treatment (30 follicles per treatment × five replicates = 150 follicles), totaling 600 preantral follicles.

For assessment of follicular activation, only morphologically normal follicles with a visible oocyte nucleus were recorded, and the proportion of primordial and growing follicles (intermediate, primary and secondary) was calculated at day 0 (fresh control) and after 7 days of culture. Furthermore, from the basement membrane, the major and minor axes of each follicle and oocyte were measured using the Motic Image Plus 5.0^®^ software (Motic China Group Co., Ltd), and the average of these two measurements was used to determine the diameters of the follicle and the oocyte, respectively.

### Immunohistochemistry

Immunohistochemical analyses of PCNA and the proinflammatory cytokine TNF-α were conducted to assess proliferating cells and inflammation, respectively. Immunohistochemistry was performed as previously described (Monte et al., 2021). Briefly, ovarian sections (at 5 µm thick) were mounted on Starfrost glass slides (Knittel, Braunschweig, Germany), and incubated with citrate buffer at 95ºC in a deckloaking chamber (Biocare, Concord, CA, USA) for antigen retrieval. Next, the ovarian sections were immersed in H_2_O_2_ to block endogenous peroxidase activity and incubated with 1% normal goat serum to block non-specific binding. Subsequently, the sections were incubated in a humidified chamber for 45 min at room temperature with rabbit polyclonal anti-PCNA (1:300) and anti-TNF-α (1:100) antibodies. Then, the sections were incubated with EasyLink One Polymer, followed by DAB staining for protein immunolocalization, and counterstaining with hematoxylin. A no primary antibody control was performed by incubating the tissues with buffer, omitting the primary antibody. Positive control for PCNA was performed using mouse testicular tissue (Chen et al., 2017).

For immunohistochemical analysis, only follicles containing a visible oocyte nucleus were analyzed using a light microscope (Nikon) connected to a computer equipped with Motic Images Plus 5.0^®^ software. The number of PCNA-positive granulosa cells (approximately 120) was counted in 10 random fields per treatment. Cells exhibiting a brown staining were considered positive, regardless the intensity. The percentage of PCNA-positive cells was calculated as the number of proliferating cells out of the total number of cells (x100). To assess inflammatory activity, the immunostaining of TNF-α (brown staining) in approximately 20 preantral follicles for each treatment was subjectively classified based on staining intensity as absent, weak, moderate, or strong (Silva et al., 2023).

### Statistical analysis

Statistical analysis were performed using BioEstat 5.0 (Barros et al., 2019). The data from morphologically normal (survival), primordial and growing follicles (activation) and cell proliferation were compared using Chi-square test and expressed as percentages. The data of follicular and oocyte diameters were evaluated using the Shapiro–Wilk test to verify the normal distribution of residues. Thereafter, the data were subjected to ANOVA and Tukey’s test for comparisons among treatments. The results were expressed as the mean ± SEM. The differences were considered statistically significant when P<0.05.

## Results

### Follicular morphology, activation and growth

**Figure 1:**
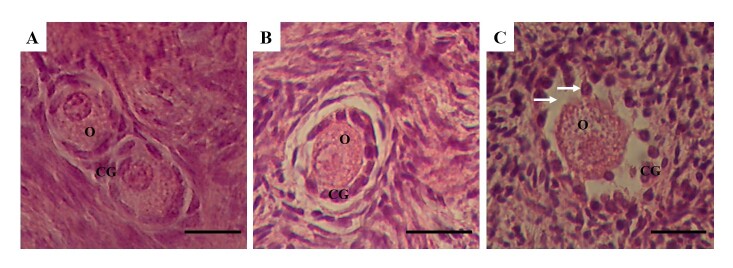
Histological sections of ovine ovarian fragments: normal preantral follicles from the fresh control (A) and after *in vitro* culture in medium containing 10 ng/mL visfatin (B), and an atretic follicle after culture in α-MEM^+^ (C). Arrow indicates signs of degeneration; O: oocyte; GC: granulosa cells. Scale bars: 30 µm (400x).

**Figure 2:**
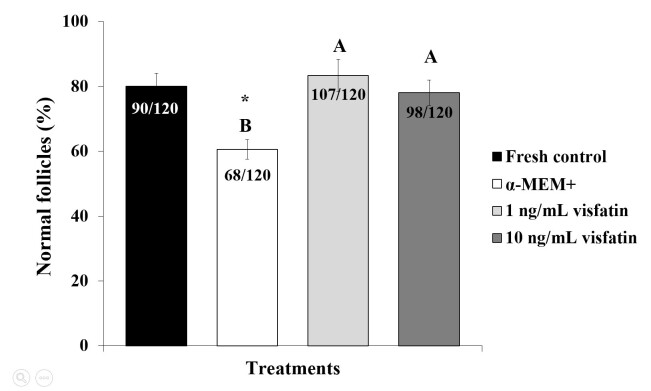
Percentages of morphologically normal follicles in the fresh control and after *in vitro* culture of ovine ovarian tissue in α-MEM^+^ or 1 or 10 ng/mL visfatin (). (*) Differs significantly from fresh control (P<0.05). (^A, B^) Different letters denote significant differences among treatments (P<0.05).

Among the preantral follicles analyzed, 138 were primordial, 275 were intermediate, 66 were primary and 1 was secondary. Findings from the histological analysis indicated that preantral follicles from fresh control (Figure 1A) or those cultured in the presence of 10 ng/mL visfatin (Figure 1B) showed morphologically normal oocytes surrounded by well-organized granulosa cells. However, follicles with a retracted oocyte and disorganized, swollen granulosa cells were commonly observed after *in vitro* culture in α-MEM^+^ (Figure 1C). According to Figure 2, after 7 days of culture, treatments containing 1 or 10 ng/mL visfatin had the same percentage of normal follicles (83.3% and 78%, respectively) as the fresh control (80%) (P>0.05) and significantly higher percentage than α-MEM^+^ (60.6%).

**Figure 3:**
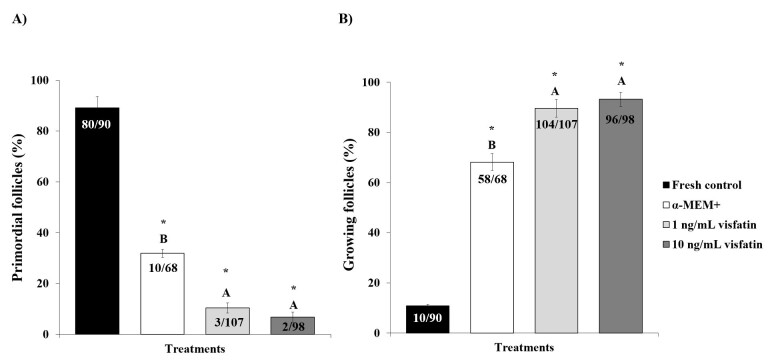
Percentages of normal primordial (A) and growing (B) follicles in the fresh control and after *in vitro* culture of ovine ovarian tissue in α-MEM^+^ or 1 or 10 ng/mL visfatin. (*) Differs significantly from fresh control (P<0.05). (^A, B^) Different letters denote significant differences among treatments (P<0.05).

After culture, there was a decrease in the percentage of primordial follicles (Figure 3A) and an increase in the percentage of growing follicles (Figure 3B) in all treatments compared to the fresh control (P<0.05). Furthermore, medium containing visfatin (1 or 10 ng/mL) exhibited greater (P<0.05) primordial follicle activation than control medium (α-MEM^+^). Both follicle and oocyte diameters increased (P<0.05) when tissues were cultured in medium containing 10 ng/mL visfatin compared to α-MEM^+^ (Table 1).

**Table 1:** Follicular and oocyte diameters (mean ± SEM) in fresh control and after *in vitro* culture of sheep ovarian tissue in α-MEM^+^ or visfatin.

**Treatments**	**Follicular diameter (µm)**	**Oocyte diameter (µm)**
Fresh control	59.24 ± 5.52	46.20 ± 4.22
α-MEM^+^	52.94 ± 5.28 B	38.70 ± 4.13 *^B^
1 ng/mL visfatin	56.44 ± 4.51 ^AB^	39.89 ± 5.51 ^AB^
10 ng/mL visfatin	63.57 ± 5.14 A	46.34 ± 4.67 ^A^

(*) Differs significantly from fresh control (P<0.05). (^A, B^) Different letters denote significant differences among treatments (P<0.05).

### Immunostaining for PCNA and TNF-α

**Figure 4:**
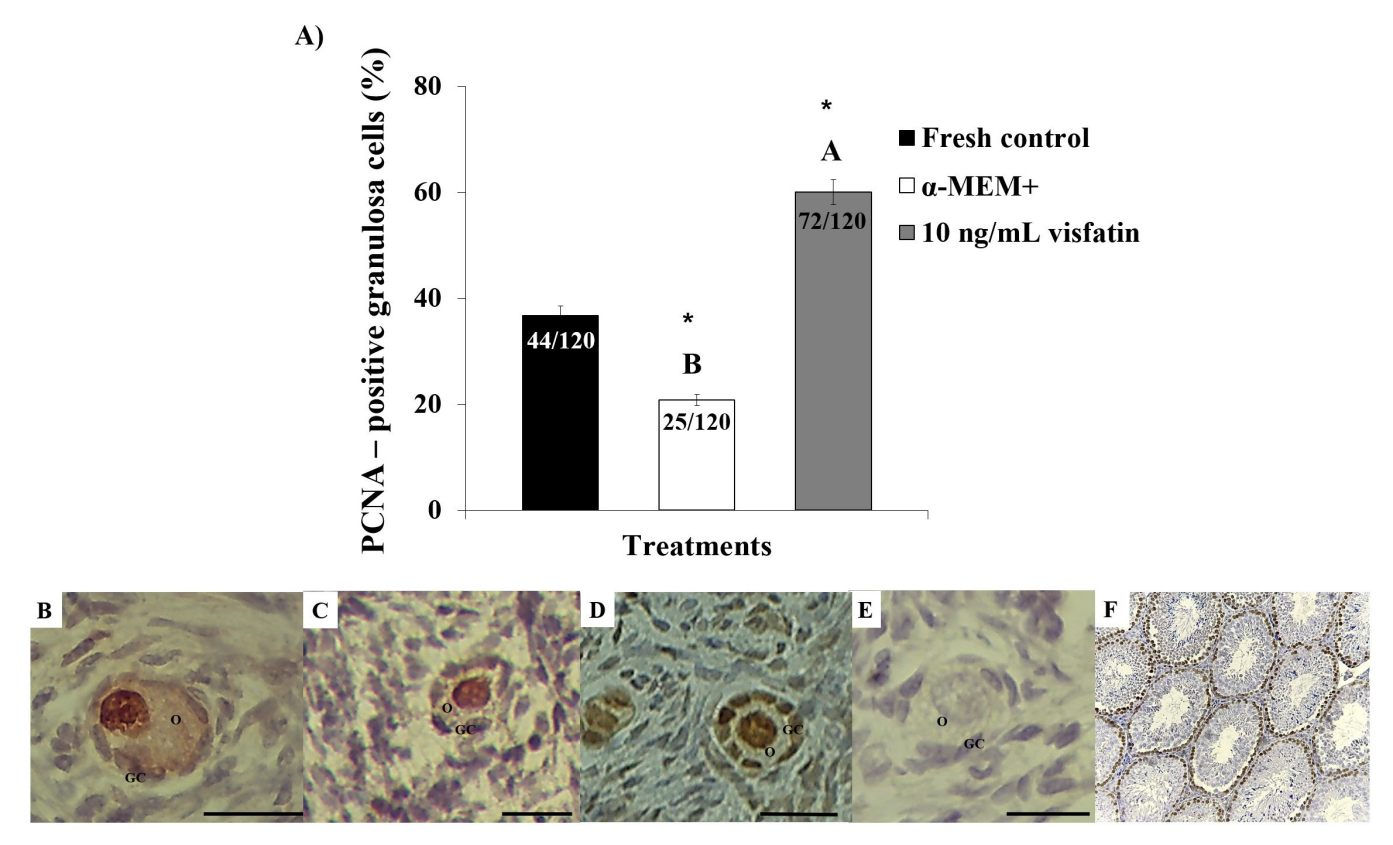
Percentages of PCNA-positive granulosa cells in fresh control and after *in vitro* culture of ovine ovarian tissue in α-MEM^+^ or in medium containing 10 ng/mL visfatin (A). (*) Differs significantly from fresh control (P<0.05). (^A, B^) Different letters denote significant differences among treatments (P<0.05). PCNA immunohistochemical: preantral follicles from fresh control (B) and after *in vitro* culture in α-MEM^+^ (C) or in medium containing 10 ng/mL visfatin (D). Control of the immunohistochemical reaction (E) and positive control (F). O: oocyte; GC: granulosa cells. Scale bars: 30 µm (400x).

Immunohistochemistry was performed on the fresh control, control medium and medium containing 10 ng/ml visfatin (the treatment that exhibited higher follicular and oocyte diameters compared to the control medium). After *in vitro* culture, the percentage of PCNA-positive granulosa cells in medium containing 10 ng/mL visfatin (60%) was significantly higher than that in the fresh control (36.66%) and α-MEM^+^ (20.83%) (Figure 4A-D). The reaction of positive controls (incubated with protein blocker) are showed in the Figures 4E and 4F, respectively.

**Figure 5:**
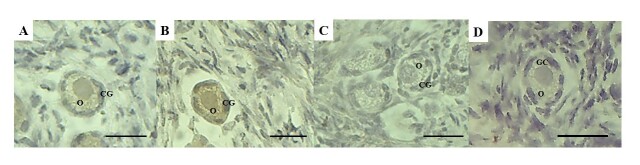
TNF-α immunostaining: preantral follicles from control group follicle (A) and after *in vitro* culture in α-MEM^+^ (B) or in medium containing 10 ng/mL visfatin (C). Control of the immunohistochemical reaction (D). O: oocyte; GC: granulosa cells. Scale bars: 30 µm (400x).

Immunohistochemical analysis for TNF-α showed weak staining in preantral follicles from the fresh control (Figure 5A), while moderate immunostaining was observed in preantral follicles cultured in control medium (Figure 5B). Culture with 10 ng/mL visfatin attenuated cell inflammation, showing minimal or no staining for TNF-α (Figure 5C). The reaction control did not show any background staining (Figure 5D).

## Discussion

The present study, for the first time, evaluated the effects of visfatin on the *in vitro* culture of sheep ovarian tissue. The results showed that the medium containing visfatin improves follicular survival by reducing TNF-α immunostaining (a pro-inflammatory cytokine) and stimulates primordial follicle activation and granulosa cell proliferation.

Regardless of the concentration tested, *in vitro* culture in medium containing visfatin resulted in similar percentages of normal follicles compared to the fresh control and higher percentages than the control medium (α-MEM^+^). Visfatin administration to aged mice increased the total number of surviving follicles (primordial, primary, secondary, and antral follicles) compared to the group not treated with visfatin (Park et al.*,* 2020). Additionally, *in vitro* culture of mice uterus and ovaries in the presence of a specific inhibitor (FK866) for endogenous visfatin resulted in a reduction in the levels of antioxidant enzymes (superoxide dismutase and glutathione peroxidase) and/or expression of anti-apoptotic marker (Bcl-2), leading to cell death by apoptosis (Annie et al.*,* 2019; Annie et al.*,* 2020).

*In vitro* conditions can lead to cell death by inducing inflammation and oxidative stress (Ding et al., 2020; Rowley et al., 2020). In the present study, culture with 10 ng/mL visfatin reduced the TNF-α immunostaining, a pro-inflammatory cytokine that contributes to the apoptosis process within the ovary (Liu et al., 2021). In *in vivo* and *in vitro* approaches of ischemia/hypoxia, visfatin effectively reduced the expression of inflammatory factors, such as interleukin-1β (IL-1β) and TNF-α, and further apoptosis of myocardial cells in rat model (Xin et al., 2020). Furthermore, visfatin helps to downregulate TNF-α biosynthesis, which prevents inflammatory processes (Moschen et al., 2010). Because exogenous visfatin may penetrate the plasma membrane by passive diffusion (Park et al., 2011; Adya et al., 2012), in the current study, we propose that visfatin directly affects cellular components, reducing inflammatory activity by lowering TNF-α immunostaining and likely decreasing oxidative stress and subsequent apoptotic events, thereby supporting follicle survival.

In our study, visfatin increased the activation of primordial follicles (90% of growing follicles) compared to the fresh control (10% of growing follicles) and α-MEM^+^ (68% of growing follicles). Furthermore, there were additive effects on follicular and oocyte growth, as well as granulosa cell proliferation, when ovarian fragments were cultured in 10 ng/mL visfatin compared to the control medium. Results from previous studies have demonstrated that exogenous visfatin increases DNA synthesis and promotes the proliferation of rat cardiac fibroblasts (Yu et al.*,* 2010), human endothelial cells (Adya et al., 2008; Adya et al., 2009) and human smooth muscle cells (Wang et al., 2009). In addition, visfatin has a stimulatory effect on the secretion of angiogenic factors, such as vascular endothelial growth factor (VEGF) and stromal cell-derived factor 1 (SDF-1α), from ovarian stromal cells and granulosa cells *in vitro* (mouse: Park et al.*,* 2020). These intraovarian factors may play an important role in follicular growth and development (goat: Araújo et al., 2011; mouse: Park et al., 2020). Therefore, visfatin is likely acting on primordial follicle activation by stimulating the proliferation of granulosa cells and further secretion of other stimulatory factors, followed by the growth of the oocyte and follicle.

Previous study demonstrated that visfatin increases IGF-1-induced steroidogenesis and cell proliferation after *in vitro* culture of human granulosa cells (Reverchon et al., 2013). Moreover, study performed by our team evidenced the important roles of IGF-1 in primordial follicle activation and granulosa cell proliferation in ovine species (Bezerra et al., 2018a). Thus, further studies will be of interest to clarify the effects of visfatin associated with IGF-1 on *in vitro* culture of preantral follicles.

Although the addition of visfatin to the culture medium at a low concentration (1 ng/ml) maintained survival and promoted follicle activation, there was no additional beneficial effect on follicular and oocyte diameters, which remained similar to the control medium. Furthermore, the lowest cell proliferation and reduced oocyte diameter found in the control medium could be explained by possible DNA damage (ovine: Bezerra et al., 2018b; Santos et al., 2019) and likely reduction in the mitosis rate of granulosa cells associated with a decreasing in the oocyte size (caprine: Costa et al., 2014), which are widely observed in preantral follicles cultured in α-MEM+.

## Conclusion

Visfatin (10 ng/mL) maintains survival, attenuated inflammatory activity by reducing TNF-α immunostaining and promotes activation and growth of primordial follicles by stimulating granulosa cell proliferation after *in vitro* culture of ovine ovarian tissue. The study *in vitro* culture of ovarian tiussue represents an important approach to maximaxe the pool of growing follicles and providing a large supply of mature female germ cells in sheep species and other economically attractive animals, and to decipher the effects of different agents on ovarian function. Future studies should focus on the mechanisms of action, including signaling pathways, by which visfatin acts on ovarian cells.

## Data Availability

Research data is only available upon request
